# Optimizing Workflow for Cone Beam Computed Tomography-Based Online Adaptive Radiation Therapy Toward Reduced Physician Involvement

**DOI:** 10.1016/j.adro.2025.101874

**Published:** 2025-07-25

**Authors:** Goda Kalinauskaite, Luise A. Künzel, Anne Kluge, Kerstin Rubarth, Jakob Dannehl, Celina Höhne, Marcus Beck, Daniel Zips, Carolin Senger

**Affiliations:** a Charité – Universitätsmedizin Berlin, corporate member of Freie Universität Berlin and Humboldt-Universität zu Berlin, Department of Radiation Oncology and Radiotherapy, Augustenburger Platz 1, 13353 Berlin, Germany; b Berlin Institute of Health at Charité – Universitätsmedizin Berlin, Charitéplatz 1, 10117 Berlin, Germany; c German Cancer Consortium (DKTK) partner site Berlin, Charité – Universitätsmedizin Berlin, Berlin, Germany; d German Cancer Research Center (DKFZ), Heidelberg, Germany; e National Center Tumor Diseases (NCT) partner site Berlin, Berlin, Germany; f Charité – Universitätsmedizin Berlin, corporate member of Freie Universität Berlin and Humboldt-Universität zu Berlin, Institut of Biometry and Clinical Epidemiology, Charitéplatz 1, 10117 Berlin, Germany

## Abstract

**Purpose:**

To evaluate the impact of an optimized online adaptive radiation therapy workflow on physician involvement.

**Methods and Materials:**

Data from a prospective phase 2 trial involving 34 prostate cancer patients treated with cone beam computed tomography (CBCT)-based online adaptive radiation therapy (62 Gy in 20 fractions) were analyzed. Manual interventions were required for 2 steps in the workflow: radiation therapy technologist review and adjustment of automatically segmented organs, guiding target segmentation, so-called “influencer,” while physicians reviewed and refined the targets. Three different workflows were compared: 2-influencer (rectum and bladder), 3-influencer (+prostate), and 5-influencer (+seminal vesicles and bowel). Time for workflow steps, extent of manual corrections, and target volume changes were compared.

**Results:**

A total of 613 fractions were analyzed. The 5-influencer workflow reduced manual target corrections to 11% of fractions compared with 51% for the 3-influencer workflow and 61% for the 2-influencer workflow (*P* < .001). Median session duration across workflows was 24.0 minutes (IQR, 22.0-28.0). Median target review times were shortest with the 5-influencer workflow at 2.5 minutes compared with 5.0 minutes for the 3-influencer workflow (*P* < .001) and 5.6 minutes for the 2-influencer workflow (*P* = .002). Most patients (84%) found the treatment time well tolerable.

**Conclusions:**

This study of prostate cancer patients suggests that optimized workflow reduces the need for physician involvement in online CBCT guided adaptive radiation therapy. Optimized workflows may facilitate a more radiation therapy technologist-driven approach similar to standard image guided radiation therapy. Further studies in other cancers, also focusing on clinical endpoints, are needed to further improve CBCT guided online adaptive radiation therapy.

## Introduction

Online adaptive radiation therapy (oART) enhances treatment precision by systematically monitoring treatment progress and dynamically adapting to changes in patient or tumor anatomy throughout the course of therapy.^1^ This approach challenges the conventional paradigm, which assumes that anatomy remains largely constant throughout treatment and that discrepancies can be addressed solely with planning margins.^2^ Daily on-couch imaging in oART accommodates interfractional anatomic variations due to organ filling, weight loss, positioning shifts, and tumor shrinkage.^3^, ^4^, ^5^ Studies of prostate, bladder, and cervical cancer have demonstrated that oART can improve target coverage while reducing dose exposure to surrounding tissues, thereby optimizing the therapeutic ratio.^3^^,^^6^^,^^7^ The integration of cone beam computed tomography (CBCT) and artificial intelligence (AI) has made CBCT-based oART widely available, likely ushering radiation therapy into a new paradigm.^8^

Despite advancements, oART implementation faces challenges. A primary concern is the time-consuming nature of the oART process. Like all previous steps in the implementation of image-guided radiation therapy (IGRT) for patients with prostate cancer, the complexity of oART further increases the in-room time. Both CBCT and orthogonal x-ray systems require more time than the conventional patient setup using wall lasers.^9^ CBCT-based oART treatment times vary by tumor location, ranging from 25 to 45 minutes, far exceeding the typical under 10 minutes of IGRT.^10^, ^11^, ^12^, ^13^

Another concern is the substantial human resources required for the oART workflow, involving radiation oncologists, medical physics experts, and radiation therapy technologists (RTTs).^12^, ^13^, ^14^ The high resource demand of oART raises concerns about its sustainability for radiation therapy departments, affecting both short- and long-term workforce dynamics.^15^ In the short term, it requires more highly trained personnel. Long-term adoption may require major investments in equipment, training, and workflow restructuring, reshaping the roles and responsibilities within radiation therapy teams while limiting scalability and research opportunities.

Optimizing the oART workflow is essential, particularly for common indications like primary prostate cancer. Streamlining the process could enhance time efficiency and reduce the extent of required human intervention, making it feasible to treat a larger patient population with oART. Optimizing oART workflows may not only benefit clinical teams and research but also enhance the patient experience.

This study aimed to optimize the CBCT-based oART workflow for prostate cancer by comparing different adaptive workflows and testing both structure guided deformation algorithms and automated target generation methods. We focused on reducing physician intervention and treatment time while maintaining accuracy. By comparing 3 different oART workflows, we sought to identify the most robust approach, assess human involvement, and enhance time efficiency. Our ultimate goal was to develop an automated oART workflow primarily supervised by RTTs akin to conventional IGRT, streamlining processes and supporting broader adoption.

## Methods and Materials

This phase 2 observational study was approved by the local ethics committee and registered on ClinicalTrials.gov (NCT06116019). We present data from the first 10 months of CBCT guided oART using the ETHOS therapy system (Varian Medical Systems/Siemens Healthineers) from January to October 2024. The study population comprised 34 patients with histologically confirmed prostate cancer of any risk group undergoing definitive radiation therapy of the prostate without lymph node treatment. Patients were excluded if they were unable to remain still during the procedure, had hip implants, or were unable to give informed consent.

### Treatment planning

Radiation therapy was delivered in 20 fractions using a simultaneous integrated boost (SIB), with 3 dose levels (62 Gy/57.6 Gy/48 Gy) prescribed to achieve D98% (dose covering 98% of the planning target volume [PTV]) ≥ 95% of the prescribed dose.^16^^,^^17^ Planning computed tomography (CT) (2 mm slices, SOMATOM go.Open Pro, Siemens Healthineers) was performed after ensuring comfortable bladder filling and rectal emptying. Organs at risk were auto-contoured using the AI-based software Annotate (ART-Plan, TheraPanacea). Clinical target volume (CTV) delineation was performed using the Eclipse v.17.0 (Varian Medical Systems/Siemens Healthineers) treatment planning system. Three CTVs were defined: CTV_62 (prostate was expanded with part of the involved seminal vesicles [SV] for cT3b), CTV_57.6 (CTV_62 was expanded with 0.5 mm margins, excluding the rectum + the base of SV for cT3b), and CTV_48 (CTV_57.6 was expanded with a distal 2 cm [cT1-3a] or complete [cT3b] SV).^16^ PTVs were created by adding a 5 mm margin to the CTVs in all directions, except 3 mm posteriorly.

For treatment planning, Clinical Directive Templates within the ETHOS Treatment planning v1.1 were used to define dose and fractionation schedules, dose-volume goals for targets and organs at risk, and instructions for deriving CTVs and PTVs, as well as the number of influencers (Table E1). All plans were generated using the ETHOS inherent Intelligent Optimization Engine.^18^ Intensity modulated radiation therapy with 9 or 12 fields was chosen due to its faster optimization and calculation times within the adaptive workflow.

### Online adaptive workflow

**Figure 1:**
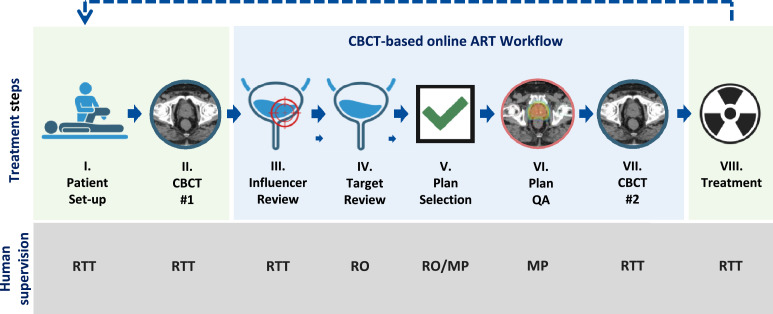
Workflow of cone beam computed tomography (CBCT)-based online adaptive radiation therapy (ART). Steps III to VII represent the procedure specific to online ART only, while steps I, II, and VIII apply to both conventional image guided radiation therapy and online ART. *Abbreviations:* MP = medical physics expert; QA = quality assurance; RO = radiation oncologist; RTT = radiation therapy technologist.

The workflow consisted of the following steps (Fig. 1):

#### Patient setup (I) and acquisition of CBCT 1 (II)

Patients were asked to ensure comfortable bladder filling prior to treatment. Except for the first 3 patients, where conventional CBCT was used, all patients were imaged with HyperSight CBCT (Varian Medical Systems/Siemens Healthineers).

#### Influencer review (III)

“Influencer” is an ETHOS-specific term referring to organs that directly influence the position and shape of the CTVs and guide deformable image registration for generating CTVs.^18^ Influencers are automatically segmented using AI and subsequently reviewed and manually corrected, if necessary, by 1 of 3 experienced and vendor-trained RTTs.

We tested a 2-influencer (bladder and rectum), 3-influencer (+prostate), and 5-influencer (+SV and ±small bowel) workflow (Table 1). Initially, the small bowel was an influencer only if near the prostate on the planning CT, but was later included for all patients due to its variable location across fractions. Influencer corrections were categorized as no, minor, intermediate, or major.^19^ We sequentially evaluated 3-, 2-, and 5-influencer workflows under routine clinical conditions to minimize physician involvement.

**Table 1:** Comparison of the 2-, 3-, and 5-influencer workflows

Workflow	Influencers	Target generation	Manual target corrections
2-influencer	Bladder and rectum	Structure guided deformation algorithm	Yes
3-influencer	Bladder, rectum, and prostate	Structure guided deformation algorithm	Yes
5-influencer	Bladder, rectum, prostate, seminal vesicles (± small bowel)	Automatically derived using margins and Boolean operations	No (must return to influencer corrections)

#### Targets review (IV)

In the 2- and 3-influencer workflows, targets were generated using the structure guided deformation algorithm^18^ and reviewed by 1 of 2 radiation oncologists responsible for adaptive treatments, with manual corrections classified like influencer edits. In the 5-influencer workflow, CTVs were automatically derived from the prostate and SV using margins and Boolean operations. Manual CTV corrections were not possible in this workflow; instead, repeated modifications to the influencers (eg, prostate and SV) had to be performed by a radiation oncologist. This process was distinct from the initial influencer review step and effectively served as a target correction.

#### Plan selection (V)

A synthetic CT, generated via deformable registration, provided electron density and Hounsfield unit data for dose calculations. Its plausibility was verified by assessing body contour and high-density structures. Plan computation began during target review but restarted if targets (2-/3-influencer) or influencers (5-influencer) were adjusted. Two plans were then presented: an adaptive plan, newly optimized for the anatomy of the day, and a scheduled plan, which is the reference plan recalculated using the synthetic CT. Selection was based on predefined dose-volume constraints (Table E1).

#### Plan quality assurance (VI)

Online plan quality assurance (QA) was conducted using Mobius3D (Varian Medical Systems), a software tool for secondary dose calculations. All generated plans were automatically transferred to Mobius3D, and secondary dose computation calculations started automatically. Plan quality was deemed adequate if at least 95% of voxels passed the global gamma criterion of 3%/2 mm.

#### Acquisition of CBCT 2 (VII) and treatment (VIII)

A second CBCT was performed just before treatment to verify anatomy—an optional but useful step given the adaptive workflow duration. If visible anatomic changes occurred within the PTV, we recorded it in our database as “yes”; otherwise, it was marked as “no”. If the changes were judged unacceptable for treatment, the workflow was restarted from the beginning. Adaptive treatment began only after plan approval by a radiation oncologist and a medical physics expert.

### Data collection and analysis

During each treatment session, data on workflow efficiency were prospectively collected. This included the time required for each workflow step, the extent of manual corrections (number of corrections and categorized by severity) for influencer and target, visible anatomic changes observed in CBCT 2, reasons for session interruptions, and changes in target volumes. These data were subsequently used as input for optimizing the workflow. Patient experience was assessed once weekly using a 19-item questionnaire adapted from Barnes et al,^20^ which covers physical discomfort, situational coping, psychological coping, and informational needs. For the current study, we analyzed only 1 item, “I found the time taken for the treatment easy to tolerate,” with response options of “Not at all,” “Slightly,” “Moderately,” and “Very.”

This analysis focuses on the following exploratory endpoints to assess workflow feasibility and optimization: the number and extent of required manual corrections for influencers and targets; time efficiency, measured as total session duration (minutes) divided into workflow steps; and changes in the volume of adaptive CTVs compared with the CTVs on the planning CT. The results are presented as exploratory and should be interpreted as hypothesis-generating.

Data were analyzed using descriptive statistical methods. The extent of required manual corrections was described as the relative frequency of all fractions. Times are provided as median and IQR. Differences among the 3 workflows were evaluated in the patient mean values using the Kruskal-Wallis test and Dunn's post hoc test with Bonferroni-Holm correction, as the data were not normally distributed. Accordingly, the difference in contouring time between 2 small bowel dose levels was analyzed using the Mann-Whitney *U* test. Estimating correlation between parameters based on session values was assessed using either a linear mixed-effects model for continuous outcomes (times and CTV volume change) or a cumulative link mixed-effects model for categorical outcomes (correction extent and visible intrafractional changes), with the patient included as a random effect to account for within-subject correlation. Estimates are reported, including 95% CIs. For detailed model information, see Tables E2-E5. All analyses were performed using Python (version 3.10.4; packages SciPy and scikit-posthocs) and R (version 4.1.1, R Foundation for Statistical Computing; packages lmerTest and ordinal). Statistical significance was defined as *P* < .05.

## Results

Thirty-four patients received a total of 613 adaptive fractions, with adaptive plans chosen in 95% of cases. Workflow distribution was as follows: 2-influencer (n = 126, 20.6%), 3-influencer (n = 241, 39.3%), and 5-influencer (n = 246, 40.1%). Nine patients switched from 2- to 3-influencer due to excessive target corrections. Most fractions (n = 565, 92.2%) used HyperSight CBCT; only 48 (7.8%) used conventional CBCT. All 3 patients treated with conventional CBCT received treatment with the 3-influencer workflow. All adaptive plans passed Mobius3D QA.

**Figure 2:**
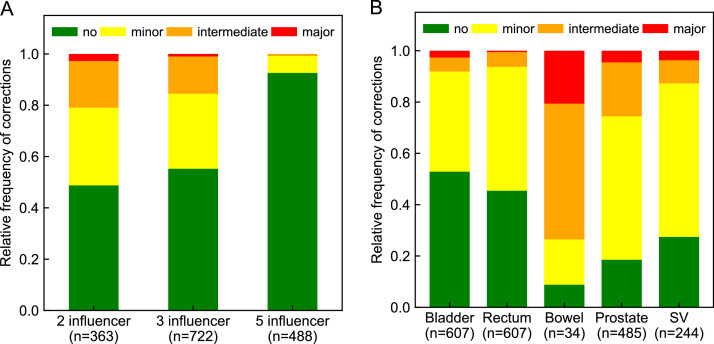
Relative frequency of manual corrections (no, minor, intermediate, and major) required for (A) targets dependent on workflow (2-, 3-, and 5-influencer) and (B) influencer structures (bladder, rectum, bowel, prostate, and seminal vesicles [SV]) independent of workflow. *Abbreviations:* n = number of fractions.

Manual target corrections (Fig. 1, step IV) were statistically significantly lower with the 5-influencer workflow (11%) compared with 3-influencer (51%) and 2-influencer (61%) workflows (*P* < .001; Fig. 2A, Table E2). The rates of influencer corrections (no, minor, intermediate, and major; Fig. 1, step III) varied by structure (Fig. 2B): bladder (53%, 39%, 5%, and 3%, respectively), rectum (45%, 48%, 6%, and 1%, respectively), bowel (9%, 18%, 53%, and 21%, respectively), prostate (18%, 56%, 21%, and 5%, respectively), and SV (27%, 60%, 9%, and 4%, respectively). Since the AI algorithm used for influencer segmentation was consistent across all workflows, we assumed that the quality of influencer segmentation did not vary and, therefore, we did not compare segmentation quality between workflows.

**Figure 3:**
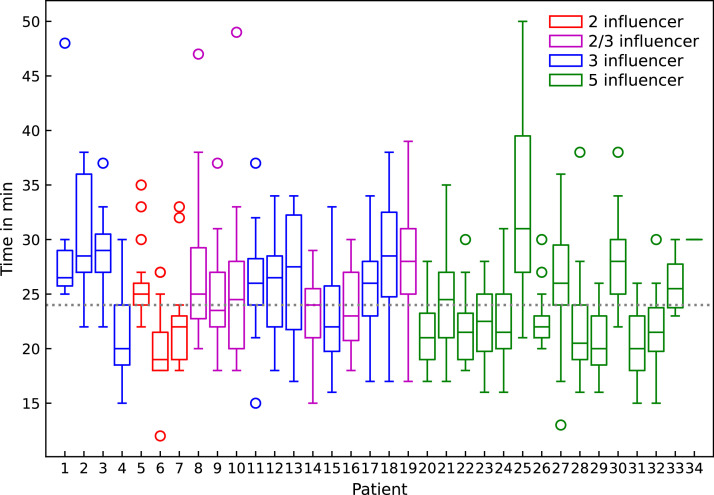
Median in-room time for all fractions (x-axis: individual patient number as sequentially treated) across the 3 workflows (2-influencer: red; 2-/3-influencer: magenta; 3-influencer: blue; 5-influencer: green. Boxes represent the interquartile range (IQR), whiskers indicate the range, and circles denote outliers. The dashed horizontal line represents the median treatment time (24.0 minutes) across all patients.

**Figure 4:**
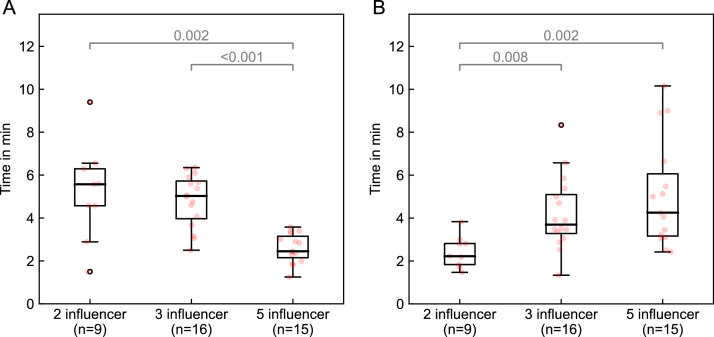
Median time needed for (A) target review (Fig. 1, step IV) and (B) influencer review (Fig. 1, step III) across the 3 workflows (2-, 3-, and 5-influencer). Red dots represent individual patient means (IQR and range). *Abbreviations:* n = number of patients.

The total median session duration (in-room time) for all fractions was 24.0 minutes (IQR, 21.0-28.0), with the treatment times per patient shown in Fig. 3 (Fig. 1, steps I-VIII). The median time required for each workflow step was as follows: influencer review (Fig. 1, step III), 3.0 minutes (IQR, 2.0-6.0); target review (Fig. 1, step IV), 3.0 minutes (IQR, 2.0-6.0); plan selection (Fig. 1, step V), 6.0 minutes (IQR, 5.0-8.0); and the combined time for the second CBCT, plan QA, and treatment delivery (Fig. 1, step VI-VIII), 6.0 minutes (IQR, 6.0-7.0). The total session time did not statistically significantly differ between the 3 workflows (*P* = .239). However, the 5-influencer workflow was faster for target review (Fig. 1, step IV), with a median time of 2.5 minutes (IQR, 2.2-3.2) compared with 5.0 minutes (IQR, 4.0-5.7; *P* < .001) for the 3-influencer workflow and 5.6 minutes (IQR, 4.6-6.3; *P* = .002) for the 2-influencer workflow (Fig. 4A). Conversely, the 2-influencer workflow was significantly faster for influencer review (Fig. 1, step III), with a median time of 2.2 minutes (IQR, 1.8-2.8) compared with 3.7 minutes (IQR, 3.3-5.1; *P* = .008) for the 3-influencer workflow and 4.3 minutes (IQR, 3.2-6.1; *P* = .002) for the 5-influencer workflow (Fig. 4B).

**Figure 5:**
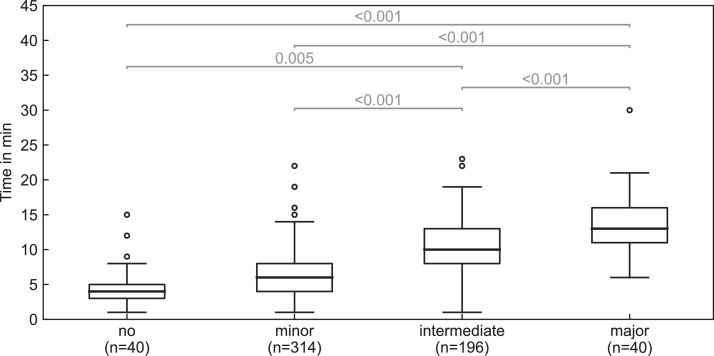
Median time (IQR and range) for the extent (no, minor, intermediate, and major) of manual corrections (Fig. 1, steps III and IV, and Table E3). *Abbreviations:* n = number of fractions.

The extent of manual corrections also influenced the time required for the adaptive session. The median time for the contouring step (Fig. 1, steps III and IV) without manual corrections was 4.0 minutes (IQR, 3.0-5.0). A linear mixed-effects model revealed significant associations between contouring time and the number of corrections (*P* < .001) as well as the extent of manual contour corrections—whether in the influencer step or target step, whichever was greatest (*P* < .001). Each additional correction of an influencer or target increased the estimated time without interactions by about 0.7 minutes (95% CI, 0.4-0.9). With regard to the extent of the corrections, the times compared with no corrections remained stable for minor corrections, while intermediate corrections increased the time by 2.2 minutes (95% CI, 0.3-4.1) and major corrections by 5.6 minutes (95% CI, 3.3-7.9) (Fig. 5, Table E3).

The cumulative mixed-effects model showed that with each 1-minute increase between the first and second CBCT scans, the odds of observing visible intrafractional changes within the PTV increased by a factor of approximately 1.1 (*P* = .009; Table E4).

Even when the small bowel was not reported as an influencer, in some cases, it was close to the target area. This increased the overall complexity of the treatment, which was reflected in a statistically significantly longer total treatment time of 25.0 minutes (IQR, 22.9-27.6) for patients with small bowel doses above the median (0.14 Gy/fraction) compared with 22.8 minutes (IQR, 20.9-24.9) for those below the median (*P* = .041).

The change in CTV_62 volume, corresponding to the prostate, relative to the planning CT volume, ranged from −42% to +44%. In a linear mixed-effects model analysis, a significant linear association (*P* = .014) was observed between the extent of manual contour corrections—whether in the influencer step or target step, whichever was greatest—and the CTV_62 volume change. Without any corrections, the estimated mean change in CTV_62 volume was +1.9% (95% CI, −2.3% to 6.1%). In comparison, corrections categorized as “minor,” “intermediate,” and “major” increased the CTV_62 volume change by an additional +3.2% (95% CI, 0.4%-6.1%), +6.3% (95% CI, 2.5%-10.1%), and +5.9% (95% CI, 1.1%-12.9%), respectively. Neither the initial target volume (*P* = .453) nor the fraction number (*P* = .400) showed a significant effect on the volume change (Table E5). Since the volumes of CTV_57.6 and CTV_48 were derived through expansion and Boolean operations from other structures, and only 2 workflows directly edited CTV_48 and CTV_57.6, these volumes were closely related to CTV_62 and, therefore, were not tested separately.

Patient experience data showed that the proportion of patients reporting tolerable treatment times increased over the course of therapy, rising from 86% in the first week to 96% by the final week.

## Discussion

This study analyzed 3 oART workflows with CBCT-Linac for prostate cancer, focusing on their impact on time efficiency and physician involvement. The results demonstrate that the 5-influencer workflow reduced the need for physician action, with only 11% of fractions requiring target correction—far lower than the 51% and 61% seen with the 3- and 2-influencer workflows, respectively. Thus, 89% of treatments could be handled entirely by RTTs, highlighting the workflow’s robustness. Despite the higher number of influencers, the 5-influencer workflow maintained comparable overall treatment times, with notable reductions in target review duration. These results support a shift toward an RTT-led automated workflow akin to conventional IGRT, with physicians primarily performing offline reviews.

The complex SIB approach in our study required careful evaluation of targets. Byrne et al^21^ analyzed oART with the ETHOS system and reported no manual adjustments in 72% of targets, though without using an SIB approach. Moazzezi et al^22^ found that 70% of targets in 250 simulated fractions needed only minor edits, mainly to exclude proximal SV. Another clinical study of 11 patients (7 with SIB) reported no target edits under on-site physician supervision, though all fractions required influencer adjustments.^14^ In contrast to the aforementioned studies, our 5-influencer workflow derived targets directly from prostate and SV influencers using margins and Boolean operations rather than a structure guided deformation algorithm. Even with more complex targets, our workflow demonstrates a lower^21^^,^^22^ or similar^14^ rate of manual adjustments compared with the aforementioned studies. This strategy may be especially useful for managing multilevel SIB in prostate cancer, helping streamline workflows and limiting daily physician input.

A valid criticism of oART is its time and resource intensity. One study estimated an added $103.58 per adapted fraction in the pelvic region due to increased personnel needs.^23^ In our study, influencer and target reviews (Fig. 1, steps III-IV) were the most time-consuming, comprising about one-fourth of the session. The 2-influencer workflow sped up influencer review but led to more target edits and delays in reoptimization. In contrast, the 5-influencer approach reduced target edits and physician involvement. Similar studies report influencer review times of 3 to 6 minutes (4-5 influencers) and 1 to 3 minutes for targets.^14^^,^^19^ While total in-room times did not differ significantly, most 5-influencer sessions stayed under the 24-minute median (Fig. 3). This efficiency, along with fewer target edits, underscores its potential to streamline adaptive radiation therapy and enhance patient throughput.

Despite longer in-room sessions than IGRT, oART’s median 24-minute duration was well tolerated, and tolerability rose over the course of therapy. Since in-room times were similar across workflows, we did not compare tolerability between them; a detailed oART versus IGRT analysis is forthcoming.

Changes in target volumes during radiation therapy, including the prostate, have been reported in other studies.^13^^,^^24^ This may result from variations in bladder or rectal filling, prostate size, or interobserver differences when adjusting AI-generated contours. We found that intermediate and major corrections to the prostate or CTV_62 significantly increased its volume. Whether this reflects true anatomic changes or contouring variability remains to be investigated. Well-trained RTTs are essential to maintain workflow efficiency and precision.

The need for faster workflows is highlighted by the increased risk of visible intrafractional changes with longer intervals between CBCT scans. Reducing adaptive workflow time may help prevent such changes and improve accuracy. Byrne et al^25^ reported that a 17-minute workflow allowed 4 mm PTV margins; further streamlining could potentially reduce PTV margins even further.

This study has several limitations. First, waiting times for physician input were not recorded, possibly masking workflow delays. Second, involvement of multiple clinicians may have introduced variability in contour edits, despite standardized protocols, though this reflects routine clinical practice. Third, the single-center design, use of specific technology (Varian ETHOS), and restriction to whole-organ CTVs (bladder and prostate) limit the generalizability of our findings.

## Conclusions

This study of prostate cancer patients suggests that an optimized workflow reduces the need for physician involvement in online CBCT guided adaptive radiation therapy. Optimized workflows may facilitate a more RTT-driven approach similar to standard IGRT. Further studies of other cancers, also focusing on clinical endpoints, are needed to further improve CBCT guided oART.

## Disclosures

Daniel Zips discloses financial, technical, and educational support for scientific projects received by the Department of Radiation Oncology, University of Tübingen, and the University of Charité (Elekta, Philips, Varian/Siemens, Sennewald, Therapanacea, and PTW). Presentation fees for the mentioned industrial partners were paid to the University of Tübingen and the University of Charité. Goda Kalinauskaite, Luise A. Künzel, Jakob Dannehl, Celina Höhne, Marcus Beck, and Carolin Senger disclose financial, technical, and educational support from Varian Medical Systems Inc provided to the Department of Radiation Oncology, University of Charité.

## Appendix Supplementary materials

Supplementary_material Image, application 1

## Footnotes

Supplementary material associated with this article can be found in the online version at doi:10.1016/j.adro.2025.101874.
