# Supplementary material for: Optimizing Workflow for Cone Beam Computed Tomography-Based Online Adaptive Radiation Therapy Toward Reduced Physician Involvement
Source: Adv Radiat Oncol. 2025 Jul 25;10(10):101874. doi: 10.1016/j.adro.2025.101874 (PMC12414823; doi:10.1016/j.adro.2025.101874)
Supplement: Supplementary_material [file mmc1.docx]

| **Table E1.** Used planning template for 5 influencer workflow with contoured and derived structures and assigned clinical goals, including priorities and order. Clinical goals and priorities can be fine-tuned per patient. Deviation represents the at most accepted value as internally defined for plan quality acceptance. Help structures to control dose conformality are denoted as “HS_conf”, structures denoted with “z_” are used for subsequent Boolean operations. | | | |
| --- | --- | --- | --- |
|  | **Structure** | **Origin** | **Clinical goal (deviation)** |
| **Priority 1** | Anorectum | contoured | D0.03cm³ ≤ 61.5 Gy (62.0 Gy) |
|  | PTV_62 GY | z_PTV_62 GY – z_PRV_Bowel | D98% ≥ 96% (95%) |
|  | PTV_48 GY | z_PTV_48 GY – z_PRV_Bowel | D98% ≥ 96% (95%) |
|  | PTV_57.6 GY | z_PTV_57.6 GY – z_PRV_Bowel | D98% ≥ 96% (95%) |
|  | PTV_62 GY |  | D2% ≤ 103% (105%) |
|  | HS_conf_PTV_62 GY | PTV_57.6 GY – (PTV_62 GY + 0.2 cm) | D2% ≤ 58.9 Gy (60.0 Gy) |
| **Priority 2** | Anorectum | contoured | V50 Gy ≤ 10% (22%) |
|  | Anorectum | contoured | V40 Gy ≤ 22% (38%) |
|  | Bladder | contoured | V62 Gy ≤ 3% (5%) |
|  | HS_conf_PTV_57.6 GY | PTV_48 GY – (PTV_57.6 GY + 0.3 cm) | D2% ≤ 54.7 Gy (56.0 Gy) |
|  | HS_conf_PTV_48 GY | Wall of PTV_48 GY, inner -0.4 cm, outer +2.0 cm | D2% ≤ 45.6 Gy (48.0 Gy) |
|  | Anorectum | contoured | V20G y ≤ 40% (85%) |
|  | Bladder | contoured | V48 Gy ≤ 15% (25%) |
|  | Bladder | contoured | V40 Gy ≤ 20% (50%) |
|  | Bag_Bowel | contoured | D0.03cm³ < 44 Gy (<= 46 Gy) |
| **Priority 3** | PenileBulb | contoured | V40 Gy ≤ 30% (50%) |
|  | Anorectum | contoured | V30 Gy ≤ 30% (60%) |
|  | Skin | wall of Body, inner +1.0 cm, outer -0.3 cm | D0.5 cm³ ≤ 30.0 Gy (35.0 Gy) |
|  | RectalWall | Anorectum – HS2_RectalWall | D2% ≤ 30.0 Gy (35.0 Gy) |
| **Priority 4** | Colon_sigmoid | contoured | V62 Gy ≤ 0.1% |
|  |  |  | V40 Gy ≤ 16% |
|  |  |  | V20 Gy ≤ 32% (70%) |
|  | Femur_Head_L/R | contoured | V50 Gy ≤ 5% (10%) |
|  | PenileBulb | contoured | D_Mean_ ≤ 20 Gy |
|  | GTV | contoured |  |
|  | Prostate | contoured |  |
|  | Seminal vesicles | contoured |  |
|  | Body (External) | contoured |  |
|  | CTV_48 GY | CTV_57.6 GY + HS_SB |  |
|  | CTV_57.6 GY | (Prostate + 0.5 cm) - Anorectum |  |
|  | CTV_62 GY | Prostate + 0.0 cm |  |
|  | z_Prostate +2cm | Prostate + 2.0 cm |  |
|  | z_SB | HS Prostate + 2 cm Vesicula seminalis |  |
|  | z_PRV_Bowel | Bag_Bowel + 0.1 cm |  |
|  | z_PTV_48 GY | CTV_48 GY + 0.5 cm left/right, 0.5 cm cranial/caudal, 0.5 cm anterior, 0.3 cm posterior |  |
|  | z_PTV_57.6 GY | CTV_57.6 GY + 0.5 cm left/right, 0.5 cm cranial/caudal, 0.5 cm anterior, 0.3 cm posterior |  |
|  | z_PTV_62 GY | CTV_62 GY + 0.5 cm left/right, 0.5 cm cranial/caudal, 0.5 cm anterior, 0.3 cm posterior |  |
|  | z1_RectalWall | Anorectum + 1.0 cm left/right, 1.0 cm anterior |  |
|  | z2_RectalWall | HS1_RectalWall – 0.2 cm posterior |  |

| **Table E2.** Summary of cumulative link mixed-model with formula: “Target correction extent” ~ “Workflow (reference level: “5 influencer”)” + (1 \| Patient) + (1 \| Target). | | | | | | |
| --- | --- | --- | --- | --- | --- | --- |
| **Random**  **Effects** | **Group** | **Variance** | **Standard deviation** | **Number of groups** |  |  |
|  | Patient (Intercept) | 1.85 | 1.36 | 34 |  |  |
|  | Target (Intercept) | 0.21 | 0.45 | 5 |  |  |
| **Fixed**  **Effects** | **Coefficient** | **Estimate** | **Standard error** | **Confidence interval (95%)** | **z-value** | **p-value** |
|  | Workflow \|2 Influencer | 2.41 | 0.69 | 1.07-3.75 | 3.52 | <0.001 |
|  | Workflow \|3 Influencer | 3.61 | 0.70 | 2.24-4.99 | 5.16 | <0.001 |

| **Table E3.** Summary of the linear mixed-model with formula: “Contouring time” ~ “Number of corrections” + “Extent of corrections” + Fraction + (1 \| Patient). | | | | | | | |
| --- | --- | --- | --- | --- | --- | --- | --- |
| **Random**  **Effects** | **Group** | **Variance** | **Standard deviation** | | **Number of groups** |  |  |
|  | Patient (Intercept) | 1.15 | 1.07 | 34 | |  |  |
|  | Residual (within-group) | 9.27 | 3.05 |  | |  |  |
| **Fixed**  **Effects** | **Coefficient** | **Estimate** | **Standard error** | **Confidence interval (95%)** | | **t-value** | **p-value** |
|  | Intercept | 6.55 | 0.46 | 5.65, 7.44 | | 14.26 | <0.001 |
|  | Fraction | 0.04 | 0.02 | 0.00, 0.09 | | 1.98 | 0.049 |
|  | Number of corrections | 0.66 | 0.12 | 0.42, 0.90 | | 5.39 | <0.001 |
|  | Extent of corrections \|Linear | 4.27 | 0.63 | 3.04, 5.50 | | 6.76 | <0.001 |
|  | Extent of corrections \|Quadratic | 1.71 | 0.44 | 0.86, 2.56 | | 3.93 | <0.001 |
|  | Extent of corrections \|Cubic | -0.26 | 0.27 | -0.78, 0.26 | | -0.97 | 0.331 |

| **Table E4.** Summary of cumulative link mixed-model with formula: “visible intra-fractional changes” ~ “Time between CBCT #1 and #2” + Fraction + (1 \| Patient). | | | | | | |
| --- | --- | --- | --- | --- | --- | --- |
| **Random**  **Effects** | **Group** | **Variance** | **Standard deviation** | **Number of groups** |  |  |
|  | Patient (Intercept) | 2.06 | 1.44 | 34 |  |  |
| **Fixed**  **Effects** | **Coefficient** | **Estimate** | **Standard error** | **Confidence interval (95%)** | **z-value** | **p-value** |
|  | Fraction | -0.13 | 0.04 | -0.20, -0.06 | -3.46 | <0.001 |
|  | Time CBCT#1 to #2 | 0.11 | 0.04 | 0.03, 0.19 | 2.62 | 0.009 |

| **Table E5.** Summary of the linear mixed-model with formula: “CTV_62 volume change” ~ “Extent of prostate/CTV_62 correction” + “Initial target volume” + Fraction + (1 \| Patient). | | | | | | |
| --- | --- | --- | --- | --- | --- | --- |
| **Random**  **Effects** | **Group** | **Variance** | **Standard deviation** | **Number of groups** |  |  |
|  | Patient (Intercept) | 113.3 | 10.64 | 34 |  |  |
|  | Residual (within-group) | 81.7 | 9.04 |  |  |  |
| **Fixed**  **Effects** | **Coefficient** | **Estimate** | **Standard error** | **Confidence interval (95%)** | **t-value** | **p-value** |
|  | Intercept | 9.67 | 6.24 | -2.55, 21.9 | 1.55 | 0.130 |
|  | Fraction | 0.06 | 0.07 | -0.07, 0.19 | 0.84 | 0.400 |
|  | Initial target volume | -0.08 | 0.10 | -0.27, 0.12 | -0.76 | 0.453 |
|  | Extent of corrections \|Linear | 4.65 | 1.88 | 0.98, 8.33 | 2.48 | 0.014 |
|  | Extent of corrections \|Quadratic | -1.80 | 1.38 | -4.49, 0.91 | -1.30 | 0.195 |
|  | Extent of corrections \|Cubic | -0.71 | 0.91 | -2.49, 1.07 | -0.78 | 0.434 |
